# Non-local correlation dynamics in two-dimensional graphene

**DOI:** 10.1038/s41598-022-07204-5

**Published:** 2022-03-04

**Authors:** Abdel-Baset A. Mohamed, Abdel-Haleem Abdel-Aty, Montasir Qasymeh, Hichem Eleuch

**Affiliations:** 1 Department of Mathematics, College of Science and Humanities in Al-Aflaj, Prince Sattam bin Abdulaziz University, Al-Kharj, Saudi Arabia; 2 Department of Mathematics, Faculty of Science, Assiut University, Assiut, Egypt; 3 Department of Physics, College of Sciences, University of Bisha, P.O. Box 344, Bisha, 61922 Saudi Arabia; 4 Physics Department, Faculty of Science, Al-Azhar University, Assiut, 71524 Egypt; 5 Department of Electrical and Computer Engineering, Abu Dhabi University, Abu Dhabi, 59911 United Arab Emirates; 6 Department of Applied Physics and Astronomy, University of Sharjah, Sharjah, United Arab Emirates; 7 College of Arts and Sciences, Abu Dhabi University, Abu Dhabi, 59911 United Arab Emirates; 8 Institute for Quantum Science and Engineering, Texas A&M University, College Station, TX 77843 USA

**Keywords:** Quantum information, Quantum simulation

## Abstract

We explore the non-local correlation dynamics in a Graphene sheet of disordered electrons in a two-dimensional honeycomb lattice, containing two sublattices, induced by the interaction range of impurity potentials of two Dirac points. The Bell function, uncertainty-induced non-locality, and concurrence are used to investigate the formation and robustness of the non-local correlation between the honeycomb lattice and the Dirac point. The generated lattice-point non-local correlations are explored when the lattice-point system is initially in the uncorrelated state. Due to the lattice-point interaction, the resulting Bell-function non-locality and entanglement concurrence satisfy the hierarchy principle. The generated uncertainty-induced non-locality correlation has a higher degree of stability and robustness than the Bell non-locality and concurrence. We analyze the robustness of the initial maximal non-local correlations under the effects of the band parameter, the intravalley scattering processes, the wave numbers, and the intrinsic decoherence. The formation and stability of lattice-point correlations are highly dependent on the honeycomb lattice and Dirac point characteristics.

## Introduction

Graphene is a novel carbon-based material that has the potential to be beneficial in a variety of applications, including energy storage, drug delivery, biomedical applications, sensors, electronics, membranes, and quantum applications^1–6^. The most useful aspect of graphene is that it can be made from extremely low-cost materials using a variety of chemical and physical processes, making it more suitable for commercial use^8^. Due to the innovative applications of the optical properties and long coherence time of the Graphene and Graphene compounds^7^, many promising applications such electro-optic squeezing^9^, microwave-to-optical conversion^10^, quantum teleporation^11^, and quantum memory and communications have been investigated and reported^6^.

Kozikov et al. studied the phase coherence of the Graphene spin qubits as a model of quantum memory^12^. Quantum control of the coherence and Rabi oscillations of two-qubit spin states in a Graphene is investigated in^13^. Coherent control of quantum states in monolayer Graphene interacting with a strong laser field is explored in^14^. Bound and degeneracy states for three different models of Graphene to be used as a host for spin qubits in comparison with more standard materials like GaAs were studied in^15^. Schnez et al. detected excited states in a graphene quantum dot via direct transport experiments^16^. Wu and Lue suggested graphene-based qubits in photonic quantum communications as a solid-state component of a quantum network. The valley pair qubit in gapped graphene double quantum dots is being studied as a type of quantum memory in the realization of quantum repeaters^17^. Also, the ability to generate quantum gates using graphene qubits was introduced in^18,19^. Previous studies proved that graphene is one of the most important materials that will be used in the field of quantum applications.

One of the most significant quantum resources for quantum technologies is the quantum correlation (QC)^21^. Some quantum correlated states are devoid of quantum entanglement^22,23^. As a result, various methods for detecting QC, other than entanglement, have been developed, including measurement-induced disturbance^24^, quantum discord^25^, and geometrical measures. They are based on *p*-norm^26^ and skew information quantity^27,28^. QCs can be applied in various branches of quantum engineering, quantum cryptography, and quantum information^20,29,31,32^. Quantum correlation has been extensively used to investigate the dynamic behavior of Graphene models. The quantum correlation over the two-particle quantum states of Graphene quantum dots was analyzed in^33^. The entanglement, quantum discord, and squeezing of two graphene quantum dots interacting with a thermal reservoir were computed^34,35^. In this work, the Bell function, the uncertainty-induced non-locality, and the concurrence are used to investigate the formation and robustness of the non-local correlation between the honeycomb lattice and the Dirac point. Entanglement, as an important quantum information resource, was implemented in different artificial Graphene qubit models. These models describe Bernal-stacked Graphene bilayers in the presence of trigonal warping in the energy spectrum^36^ and a tight-binding including mass and bias voltage^37^. In addition, the bilayer graphene lattice-layer entanglement was analyzed in the presence of non-Markovian phase noise^38^.

The manuscript is organized as follows: "The considered model" section introduces the theoretical model and its solution. The non-locality quantifiers of the quantum properties of the system are presented in "Non-locality quantifiers" section . The analysis of the results of the non-local correlations is illustrated in "Non-locality dynamics" section. "Conclusions" section concluded our findings.

## The considered model

Here, we consider a graphene sheet of disordered electrons in a two-dimensional honeycomb lattice. The lattice structure can be described by studying a primitive unit cell containing two sublattices (*A* and *B*). The two sites of the sublattices are denoted by up $|\uparrow \rangle$ and down $|\downarrow \rangle$ pseudospins in the representation of the Pauli matrix $\vec {\sigma }=(\sigma _{x}, \sigma _{y}, \sigma _{z})$. The two inequivalent valleys occurring at two different Dirac points are represented in another Pauli matrix $\vec {d}=(d_{x}, d_{y}, d_{z})$, which is spined by the states $|1\rangle$ and $|0\rangle$. The Hamiltonian around two Dirac points is given, within the effective-mass approximation, by^39–41^:

1 $$\begin{aligned} {\hat{H}}= & {} \eta [{\hat{n}}_{x}(\sigma _{x}\otimes {\hat{I}})+{\hat{n}}_{y}(\sigma _{y}\otimes d_{z})]+U_{A}+U_{B}, \end{aligned}$$

where $\eta$ denotes the band parameter, ${\hat{n}}_{x}$ and ${\hat{n}}_{y}$ are wave number operators, and ${\hat{I}}$ is an identity matrix. The $U_{A}$ and $U_{B}$ represent the potential of the impurities localized at *A* and *B* sublattices, respectively. They are defined as:

$$\begin{aligned} U_{A}= & {} \frac{1}{2}\lambda _{A}[({\hat{I}}+\, \sigma _{z})\otimes ({\hat{I}}+\vec {e}_{r}\cdot \vec {d})];\\ U_{B}= & {} \frac{1}{2}\lambda _{B}[({\hat{I}}- \, \sigma _{z})\otimes ({\hat{I}}+\vec {e}_{r}\cdot \vec {d})]. \end{aligned}$$

Here we focus on the case where $\lambda _{A}=\lambda _{B}=\lambda$. $\vec {e}_{r}=(\cos \alpha , \sin \alpha ,0)$ when the intravalley scattering processes are accompanied by a phase shift $\alpha$. We consider the short-range impurity potential in the standard basis of the two pseudospin states $\{|\uparrow 1\rangle , |\uparrow 0\rangle , |\downarrow 1\rangle , |\downarrow 0\rangle \}$. Hence, the energy eigenstates of the Hamiltonian of Eq. (1) are given by

2 $$\begin{aligned} \left( \begin{array}{c} |D_{1}\rangle \\ |D_{2}\rangle \\ |D_{3}\rangle \\ |D_{4}\rangle \\ \end{array} \right)= & {} \, A\left( \begin{array}{c} |\uparrow 1\rangle \\ |\uparrow 0\rangle \\ |\downarrow 1\rangle \\ |\downarrow 0\rangle \\ \end{array} \right) , \end{aligned}$$

3 $$\begin{aligned} A \,=\, & {} [a_{ij}] =\left( \begin{array}{cccc} \chi _{+} &{} \frac{1}{2}\phi &{} \frac{1}{2} &{} \phi \chi _{+} \\ -\chi _{+} &{} \frac{1}{2}\phi &{} \frac{1}{2} &{}- \phi \chi _{+} \\ \frac{1}{2} &{}- \phi \chi _{-} &{} \chi _{-} &{}-\frac{1}{2}\phi \\ \frac{1}{2} &{} \phi \chi _{-} &{} - \chi _{-} &{}-\frac{1}{2}\phi \\ \end{array} \right) , \end{aligned}$$

where the $4\times 4$-matrix *A* satisfies $AA^{\dagger }=I$ and the intravalley scattering processes are accompanied by the phase shift $\phi =e^{-i\alpha }$. The coefficients $\chi _{\pm }$ are given by:

4 $$\begin{aligned} \chi _{ \pm } & = - \frac{1}{{2\Lambda }}\left[ {\frac{\eta }{\lambda }(n_{x} \mp in_{y} \pm 1)} \right], \\ \Lambda _{ \pm } & = \sqrt {1 + \left( {\frac{\eta }{\lambda }} \right)^{2} \left( {n_{x}^{2} + n_{y}^{2} } \right) \pm 2\frac{\eta }{\lambda }n_{y} } . \\ \end{aligned}$$

The Hamiltonian eigenvalues $D_{k} (k=1,2,3,4)$, which correspond to the eigenstates $\{|D_{k}\rangle \}$, are given by:

5 $$\begin{aligned} D_{1,2}=\lambda (1\mp \Lambda _{+}), \qquad D_{3,4}=\lambda (1\mp \Lambda _{-}). \end{aligned}$$

The Milburn intrinsic decoherence model^42^ is used to explore the dynamics of the graphene sheet effects included by the intervalley interaction and the intravalley scattering processes. The Milburn model can be applied here due to the fact that in this model its assumed that on sufficiently short time steps, the system does not evolve continuously under unitary evolution but rather in a stochastic sequence of identical unitary phase changes. This assumption is required for the considered intervalley scattering of electrons from disorder sources in graphene, which is induced by potentials with extremely short interaction range like a lattice vacancy. Where, in the short time interaction range, the potential of the impurities (which is localized at *A* or *B* sublattices) and intravalley scattering can be generated^40,41^. The intrinsic decoherence effect is explored by applying the first-order approximation of the Milburn equation^42^,

6 $$\begin{aligned} \frac{d}{d t} {\hat{M}}(t)= & {} -i[{\hat{H}},{\hat{M}}(t)]-\frac{1}{2\gamma }[{\hat{H}},[{\hat{H}},{\hat{M}}(t)]], \end{aligned}$$

where *M*(*t*) is the time-dependent density matrix and $\gamma$ is the decoherence parameter. For the coherent excitation of a two-level atomic model (of a spin half particle, ${\hat{H}}=\Omega J_{x}$, with a *x*-angular momentum $J_{x}$ and a Rabi frequency $\Omega$), it is shown that the decoherence effect is apparent if $\Omega ^{2}>10^{7}\gamma$^42^. The dcoherence times for the Milburn master equation have been introduced^42–44^. The intrinsic decoherence model has been used to explore quantum information resources dynamics in several real systems as: trapped ion coupled to an optical cavity^44^, single $C_{60}$ solid state transistors^45^, polar molecules in pendular states^46,47^ as well as two superconducting charge qubit system^48^.

To find a particular analytical solution for Eq. (6), we consider that the two qubits of the honeycomb lattice and the Dirac point started with different initial uncorrelated states:

7 $$\begin{aligned} M(0)= |\uparrow 1\rangle \langle \downarrow 0|= & {} \sum _{mn} a^{*}_{m1}a_{n1}|D_{m}\rangle \langle D_{n}| \end{aligned}$$

and maximally correlated non-symmetric Bell state: $M(0)= |\varphi \rangle \langle \varphi |$,

8 $$\begin{aligned} |\varphi \rangle= & {} \frac{1}{\sqrt{2}}[|\uparrow 1\rangle +|\downarrow 0\rangle ]\nonumber \\= & {} \sum _{mn} \frac{1}{\sqrt{2}}[a^{*}_{m1}a_{n1}+a^{*}_{m4}a_{n4}]|D_{m}\rangle \langle D_{n}|. \end{aligned}$$

We can study the generation of the non-local correlations when the system starts with correlated states, as well as investigate the robustness of the initial non-local correlations against the interactions.

Using the eigenvalues $D_{k}$ ($k=1,2,3,4$) and the eigenstates $|D_{k}\rangle$, the time-dependent state of the honeycomb lattice and the Dirac point is described by the lattice-point density matrix:

9 $$\begin{aligned} {\hat{M}}(t)= & {} \!\!\!\!\sum ^{4}_{m, n = 1} \!\!\!\!\Lambda _{mn}(t) \,I_{mn}(t) \,\langle D_{m}|M(0)|D_{n}\rangle \, |D_{m}\rangle \langle D_{n}|. \end{aligned}$$

The unitary evolution $\Lambda _{mn}(t)$ and the intrinsic decoherence $I_{mn}(t)$ terms are defined by:

10 $$\begin{aligned} \Lambda _{t}= & {} e^{-i(D_{m}-D_{n})t}, \qquad I_{t}=e^{ -\frac{1}{2\gamma } (D_{m}-D_{n})^{2}t}. \end{aligned}$$

In the following sections, the non-local correlations between the honeycomb lattice and the Dirac point will be investigated using the concurrence and the local quantum Fisher information.

## Non-locality quantifiers

- **Concurrence entanglement (CE)**: Here, the concurrence is used to quantify the entanglement between the honeycomb lattice and the Dirac point. Based on the lattice-point density matrix ${\hat{M}}(t)$ of Eq. (4), the concurrence^49,50^ is defined as,
  11 $$\begin{aligned} C(t)=\max \{0,\sqrt{\lambda _{1}}-\sqrt{\lambda _{2}}-\sqrt{\lambda _{3}}-\sqrt{\lambda _{4}}\,\}, \end{aligned}$$
  where $\lambda _{1}>\lambda _{2}>\lambda _{3}>\lambda _{4}$ are the eigenvalues of the matrix: $R={\hat{M}}(t)(\sigma _{y}\otimes \sigma _{y}) {\hat{M}}(t) (\sigma _{y}\otimes \sigma _{y})$.
- **Uncertainty-induced non-locality (UIN):** Based on the skew information quantity $I({\hat{M}}(t), {\hat{K}})$^27^, the UIN of the lattice-point density matrix ${\hat{M}}(t)$ is defined by:
  12 $$\begin{aligned} U({\hat{M}}(t))= & {} \max _{K} I({\hat{M}}(t),{\hat{K}}). \end{aligned}$$
  For the density matrix ${\hat{M}}(t)$ and a local observable ${\hat{K}}$, the skew information quantity is given by:
  13 $$\begin{aligned} I({\hat{M}}(t),{\hat{K}})=-\frac{1}{2}Tr(\sqrt{{\hat{M}}(t)}{\hat{K}}-{\hat{K}}\sqrt{{\hat{M}}(t)})^{2}. \end{aligned}$$
  The amount of skew information encoded in the density operator ${\hat{M}}(t)$^27^ is employed as a measure of the information encoded. Due to its capacity to measure the non-commutativity between ${\hat{M}}(t)$ and ${\hat{K}}$, it may also be used to quantify the uncertainty of the observable ${\hat{K}}$ of the state ${\hat{M}}(t)$. The maximal skew information between the local observables ${\hat{K}}$ and ${\hat{M}}(t)$ is represented by UIN. Based on the correlation matrix $H=[h_{ij}]$ of the lattice-point state ${\hat{M}}(t)$,
  14 $$\begin{aligned} h_{ij}={Tr}\mathbf {\big \{}\sqrt{{\hat{M}}(t)}(\sigma _{i}\otimes I)\sqrt{ {\hat{M}}(t)}(\sigma _{j}\otimes I)\mathbf {\big \}}, \end{aligned}$$
  where $\sigma _{i}, i= 1,2,3$ represent the Pauli matrices, the closed form of UIN can be written as^51^:
  15 $$\begin{aligned} {U}(t)= & {} \left\{ \begin{array}{ll} 1-\lambda _{\min }(H), &{} {\vec{\mathbf{r}}}=0; \\ \\ 1-\frac{1}{|{\vec{\mathbf{r}}}|^{2}} {\vec{\mathbf{r}}}\,\,H\,\,{\vec{\mathbf{r}}}^{T}, &{} {\vec {{r}}\ne 0}, \end{array} \right. \end{aligned}$$
  where $\parallel {\vec{\mathbf{r}}}\parallel$ is the norm Bloch vector ${\vec{\mathbf{r}}}$. If the elements of the lattice-point density matrix ${\hat{M}}(t)$ (in the standard basis $\{|i\rangle \} (i=1-4)$: $\{|1\rangle =|\uparrow 1\rangle , |2\rangle =|\uparrow 0\rangle , |3\rangle =|\downarrow 1\rangle , |4\rangle =|\downarrow 0\rangle \}$) are denoted by: $z_{ij}=\langle i|{\hat{M}}(t)|j\rangle =a_{ij}+ib_{ij}$, then the vector ${\vec{\mathbf{r}}}$ is given by:
  16 $$\begin{aligned} {\vec{\mathbf{r}}}= & {} (2a_{13}+2a_{24}, \, 2b_{31}+2b_{42}, \,2z_{11}+2z_{22}-1)^{T}. \end{aligned}$$
- **Maximal Bell function (MBF)**: The maximal values of the $B_{{\max }}(t)$ were utilized as indicators of quantum non-locality^52–54^. If this function satisfies $B_{{\max }}(t)>2$, the MBF inequality is violated (i.e., $B_{{\max }}(t)-1$ identifies the MBF nonlocal correlation when it is grater than one). For the lattice-point density matrix ${\hat{M}}(t)$, the analytical expression of the $B_{\max }(t)$ is based on the correlation matrix ${T}=[t_{ij}]$^53^, where the elements $t_{ij}$ are defined by $t_{ij}=\text {Tr}\{{\hat{M}}(t)\sigma _i^{(1)}d^{(2)}_{j}\}$, $i,j=1,2,3$. The closed form of the $B_{\max }(t)$ is given by:
  17 $$\begin{aligned} B_{\max }(t)\,= 2 \sqrt{E_{1}+E_{2} }, \end{aligned}$$
  where $E_{k} (k=1,2)$ are the two largest eigenvalues of the matrix $K={T}^{\dagger }{T}$. In our work, the MBF non-local correlation is quantified by:
  18 $$\begin{aligned} B(t)&= {} {B}_{\max }(t)-1. \end{aligned}$$
  The lattice-point state has MBF non-locality when $B(t)>1$.

Following the introduction of quantum discord^25^ as a type of non-local correlation other than entanglement, several quantifiers were introduced to measure other non-local correlations, such as CE, MBF and UIN. The CE is determined by the eigenvalues of the non-Hermitian matrix *R*, the UIN by the Wigner-Yanase skew information^27^, and the MBF by the correlation matrix *T*. Because their mathematical definitions differ, each quantifier is related to a different type of non-local correlation. Physically, all kinds of quantum correlations depend on physical parameters of the considered model and satisfy the hierarchy principle. The hierarchy principle^55–57^ establishes a closer relationship between entanglement and Bell nonlocality. In this respect, all the quantum states have Bell nonlocality are entangled states, which is necessary but is not sufficient condition.

## Non-locality dynamics

**Figure 1:**
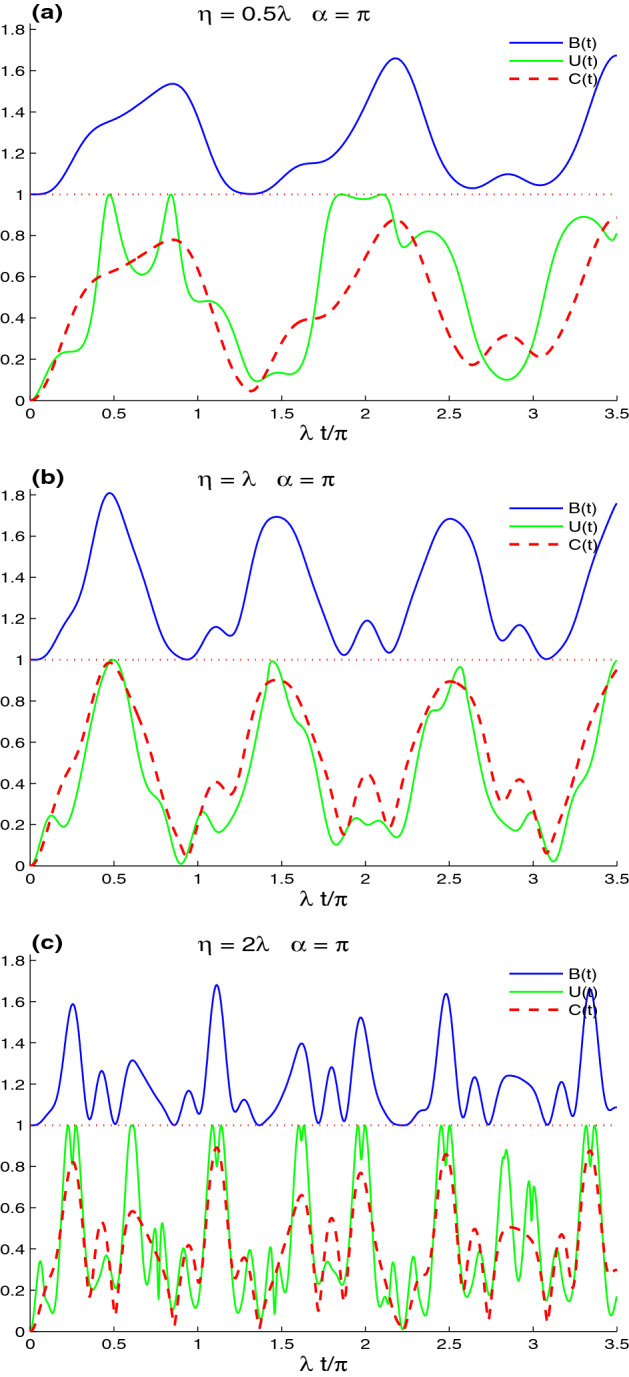
Non-local correlations of CE, UIN, and MBF for $n_{x}=n_{y}=1$, $\alpha =\pi$ and when the lattice-point system is initially in the uncorrelated state: $M(0)= |\uparrow 1\rangle \langle \downarrow 0|$ in the absence of the intrinsic decoherence with different values of band parameters $\eta$: $\eta =0.5\lambda$ in (**a**), $\eta =\lambda$ in (**b**), and $\eta =2\lambda$ in (**c**).

The generation and the robustness of the lattice-point non-local correlations of the maximal Bell function, the uncertainty-induced non-locality, and the concurrence will be here investigated under the effects of the band parameter $\eta$, and the scattering strength $\lambda$ for various wave numbers $n_{x}$ and $n_{y}$.

In the following, based on the Eqs. (4-6) and (10), we consider the scaled time $\lambda t$ which is a unitless time normalized to the scattering strength parameter. Therefore, the $\nu$ and $\gamma$ are normalized to the scattering parameter $\lambda$.

### Initial uncorrelated state

**Figure 2:**
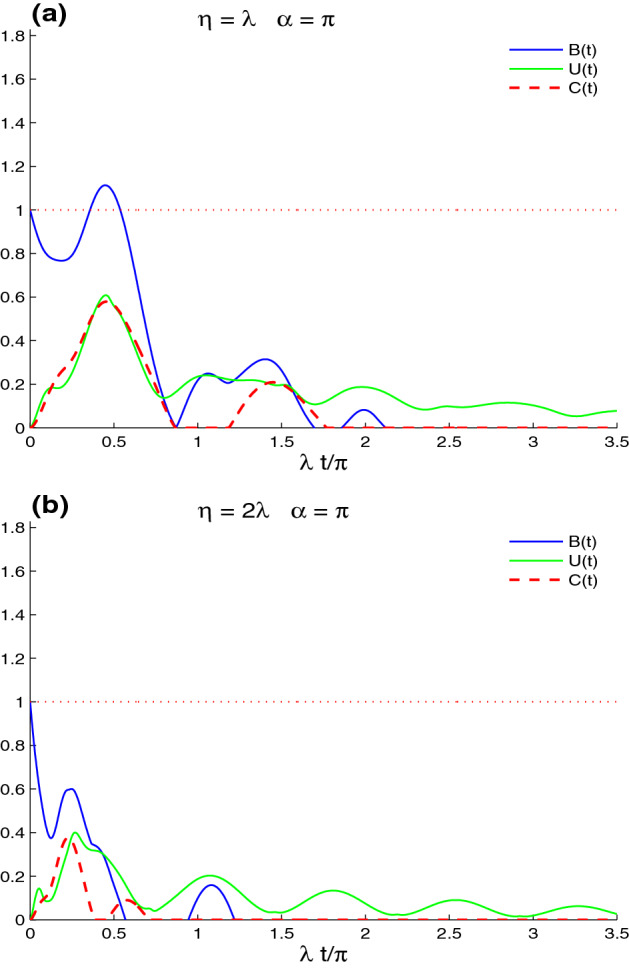
Dynamics of the nonlocal correlations of the Fig. 1b,c but in the presence of the decoherence $\gamma =0.125\times 10^{2}\lambda$.

In Fig. 1, we illustrate the generated CE, UIN, and MBF non-local correlations dynamics and their dependence on the band parameter $\eta$. Fig. 1a, displays the generation of the lattice-point non-local correlations of the Bell function, the uncertainty-induced non-locality, and the concurrence when the lattice-point system is initially in the uncorrelated state: $M(0)= |\uparrow 1\rangle \langle \downarrow 0|$ for small $\eta =0.5\lambda$. Note that the Bell-function non-locality and the entanglement concurrence are formed, due to the lattice-point interaction, and have the same oscillatory behaviour. The maximal violation of the Bell’s inequality and the MBF nonlocal correlation have always occurred. The results show that the permanent presence of the Bell-function non-locality satisfies the hierarchy principle^55–57^, as illustrated by the results. The MBF nonlocal correlation implies entanglement concurrence, which in turn implies uncertainty-induced non-locality. The lattice-point system has three different types of non-local correlations. During particular intervals, a partially lattice-point state has a maximal UIN-correlation and partial MBF non-locality.

Figure 1b shows that the increase of the band parameter $\eta$ enhances the lattice-point non-local correlations. The maximal violation intervals of Bell’s inequality increase, and the lattice-point state has MBF-nonlocality ($B(t)=2\sqrt{2}-1)$ at $t = 0.5$. In this case, the concurrence and the UIN-correlation rapidly reach their maximal correlations. The intervals of the stability of the maximal UIN-correlation are reduced. The fluctuations and the stability of the generated lattice-point non-local correlations depend on the band parameter $\eta$. For a large value of the band parameter, Fig. 1c shows that the fluctuations of the MBF nonlocal correlation, the concurrence, and the UIN-correlation rise while their amplitudes decrease, with the exception of the UIN-correlation. In this case, $\eta =2\lambda$, the uncertainty-induced non-locality presents the highest generated lattice-point non-local correlation.

**Figure 3:**
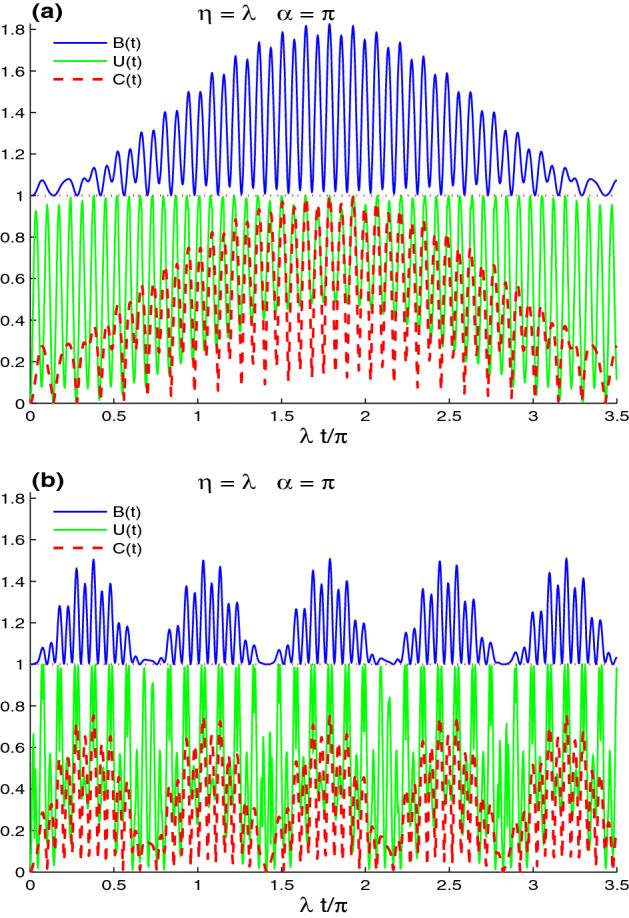
Dynamics of the non-local correlations of Fig. 1b for $(n_{x}, n_{y})=(1,7)$ in (**a**) $n_{x}=n_{y}=7$ in (**b**).

Figure 2 shows the effect of intrinsic decoherence on the dynamics of the lattice-point non-local correlations of the CE, UIN, and MBF for $\gamma =0.125\times 10^{2}\lambda$ with different band parameters’ values. Fig. 2a illustrates that the fluctuations and amplitudes of the CE, UIN, and MBF quantifiers are reduced and completely disappear after a short time. The maximal violation intervals of the Bell’s inequality vanish, except during a small interval around $t = 0.5$. For particular time windows, the generated CE presents the sudden death and sudden birth phenomena in lattice-point entanglement dynamics^58,59^. The stability and robustness of the generated UIN correlation against the high intrinsic decoherence are larger than those of the CE and MBF. In the sudden death-birth windows, the disentangled lattice-point state has a non-zero stationary UIN correlation.

Figure 2b shows that the increase of the band parameter $\eta =2\lambda$ enhances the intrinsic decoherence effect on the generated lattice-point correlations. In this case, the reduction of the fluctuations and the amplitudes of the CE, UIN, and MBF is faster than that of the case $\eta =\lambda$. UIN correlation dynamics is more robust against the decoherence. We can deduce that the generated lattice-point correlations are very fragile against the decoherence effect, with high values of the band parameter.

**Figure 4:**
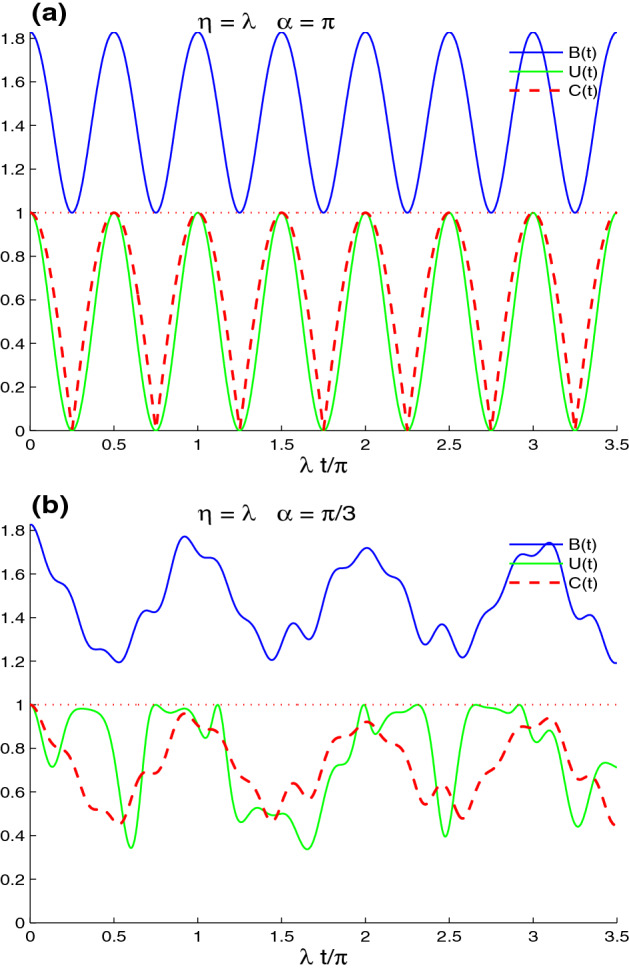
Non-local correlations for $n_{x}=n_{y}=1$, $\eta =\lambda$ and the maximally correlated state: $|\varphi \rangle =\frac{1}{\sqrt{2}}[|\uparrow 1\rangle +|\downarrow 0\rangle ]$ in the absence of the intrinsic decoherence with different values of $\alpha$: $\alpha =\pi$ in (**a**) and $\alpha =\pi /3$ in (**b**).

**Figure 5:**
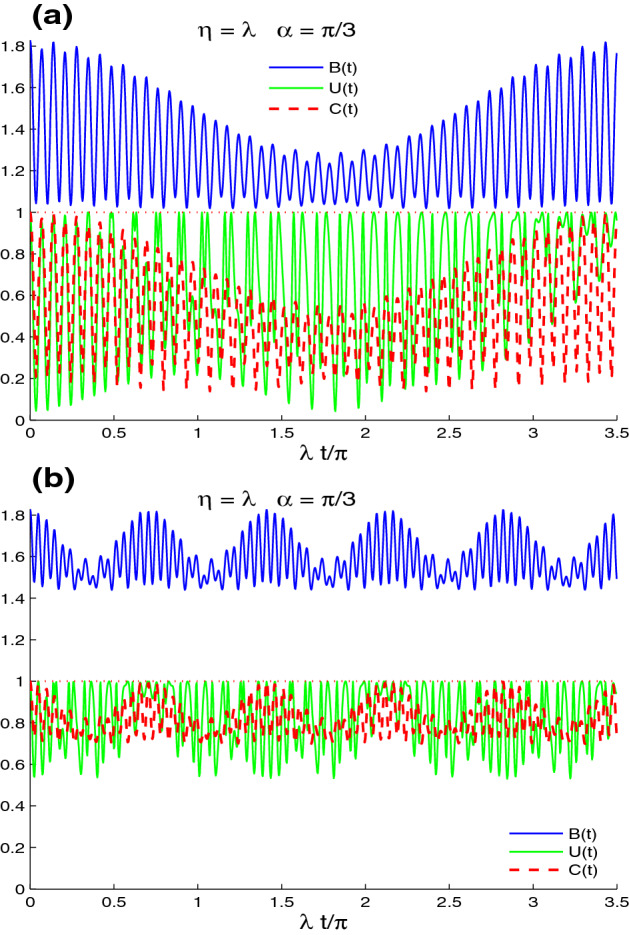
Non-local correlations for $\alpha =\pi /3$, $\eta =\lambda$ and the maximally correlated state: $|\varphi \rangle =\frac{1}{\sqrt{2}}[|\uparrow 1\rangle +|\downarrow 0\rangle ]$ in the absence of the intrinsic decoherence with $(n_{x}, n_{y})=(1,7)$ in (**a**) and $n_{x}=n_{y}=7$ in (**b**).

Figure 3 shows the dependence of the dynamics of the Bell non-locality, the UIN non-local correlation, and the concurrence entanglement on the wave numbers $n_{x}$ and $n_{y}$. Two cases are considered: $(n_{x}, n_{y})=(1,7)$ in (a), and $n_{x}=n_{y}=7$ in (b). We observe that the CE, UIN, and MBF nonlocal correlation can be controlled by varying the wave number operators. For the case $(n_{x}, n_{y})=(1,7)$, in Fig. 3a, the CE, UIN, and MBF non-local correlations grow slowly and have periodical oscillatory behaviour. We can observe that the $t_{p}$-period and the wave number operator $n_{y}$ are related by $t_{p}=\frac{n_{y}}{2n_{x}}$. They reach their maximal correlation only at the middle of the period $\frac{1}{2}t_{p}$. The dashed line in Fig. 3a, shows a rapid increase in the UIN non-local correlation with more fluctuations. The UIN reaches its maximal correlation quickly. The UIN lower bounds increase and reach maximum value at the middle of the period. For the case $(n_{x}, n_{y})=(7, 7)$, we note that the $t_{p}$-period of the periodical oscillatory behaviour for the CE, UIN, and MBF non-local correlations is reduced by increasing the wave numbers $n_{x}$ and $n_{y}$ at the same time. In each period, the fluctuations and the amplitudes of the CE, UIN, and MBF decrease. The lattice-point state has maximal UIN non-local correlation, partial CE, and MBF non-local correlations. By comparing the case $(n_{x}, n_{y})=(1, 7)$ with the case $(n_{x}, n_{y})=(7, 7)$, we find the the increasing of the energy of the second case (when the band parameter is equal to the scattering strength parameter, $\eta =\lambda$) leads to the reduction of the generated CE, UIN and MBF non-local correlations (see Fig. 3) and the preservation of the initial nonlocal correlations (see Fig. 5).

### Initial maximally correlated state

Here, we investigate the robustness of the maximal CE, UIN, and MBF non-local correlations, which are induced by considering the initial maximally correlated lattice-point state $|\varphi \rangle =\frac{1}{\sqrt{2}}[|\uparrow 1\rangle +|\downarrow 0\rangle ]$. For the initial state, the values of the CE, UIN, and MBF are $B(0)=2\sqrt{2}-1\approx 1.8284$ and $C(0)=U(0)=1$.

In Fig. 4, we illustrate the robustness of the initial maximal CE, UIN, and MBF non-local correlations against the lattice-point interaction and its dependence on the phase shift $\alpha$ of the intravalley scattering processes. In Fig. 4a, the functions *B*(*t*) *U*(*t*) and *C*(*t*) are plotted for the band parameter $\eta =1$, $\alpha =\pi$, $n_{x}=n_{y}=1$, in the absence of intrinsic decoherence. It is worth noting that the CE, UIN, and MBF functions exhibit regular oscillatory behavior, displaying the decaying and amplifying processes for the initial maximal non-local correlations with $\frac{1}{2}\pi$-period. The concurrence and the UIN present a nonlocal correlation. CE, UIN, and MBF disappear instantaneously at particular points $\frac{1}{4}(2n+1)\pi , n=0, 1, 2, ...$ (disappearance points). For the intravalley scattering phase shift $\alpha =\pi /3$, the concurrence and the MBF functions exhibit the same behavior, which differs from the UIN. The intravalley scattering processes in this situation decrease the loss of initial maximum CE, UIN, and MBF non-local correlations. These processes also prevent non-local correlations from vanishing. The UIN has a maximum value for short windows of maximum UIN-correlation stability (MCS-intervals)^60^, indicating that the UIN-correlation is more resistant to the lattice-point interaction.

**Figure 6:**
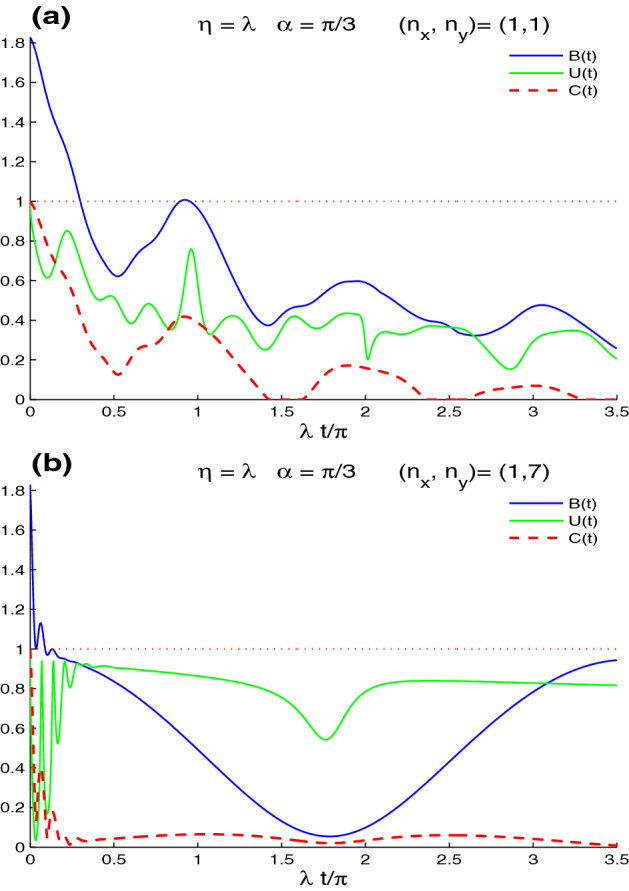
Dynamics of the non-local correlations of Figs. 4b and the 5a but in the presence of the decoherence $\gamma =0.25\times 10^{2}\lambda$.

Figure 5, shows the dependence of the robustness of the lattice-point non-local correlation dynamics on the wave numbers $n_{x}$ and $n_{y}$. The CE, UIN, and MBF functions are displayed for the two cases: $(n_{x}, n_{y})=(1, 7)$ in (a), and $n_{x}=n_{y}=7$ in (b). We observe that the CE, UIN, and MBF non-local correlation can be controlled by varying the wave number operators. Non-local correlations diminish slowly with regular oscillatory behavior with $\frac{n_{y}}{2n_{x}}$-period for the situation $(n_{x}, n_{y})=(1,7)$. The variations of the CE and MBF functions indicate that their maxima decline until the period’s middle. The UIN non-local correlation is more volatile, but more resistant to the lattice-point interaction. In the case $(n_{x}, n_{y})=(7, 7)$, the CE, UIN, and MBF functions exhibit regular oscillatory behavior with small amplitudes as the period decreases. The non-local correlation minima for CE, UIN, and MBF are increased. Because of the small amplitudes, the initial lattice-point non-local correlation is extremely resistant to the lattice-point interaction. We may conclude that increasing the wave numbers $n_{x}$ and $n_{y}$ results in preserving the initial lattice-point nonlocal correlations.

By comparing Fig. 6a and Fig. 4b, we find that the increased decoherence $\gamma =0.25\times 10^{2}\lambda$ deteriorates the amplitudes and the frequencies of the lattice-point non-local correlations. The UIN-correlation is more robust against the decoherence. For particular time windows, the phenomenon of the sudden death-birth appears in the lattice-point entanglement dynamics. The MBF non-local correlation and entanglement concurrence decrease rapidly in the case of Fig. 6b, $(n_{x}, n_{y})=(1,7)$, because the decoherence is greater than that in $n_{x}=n_{y}=1$. The UIN oscillates briefly before presenting a stable correlation that is stronger and more persistent.

## Conclusions

In the present work, we have considered a graphene sheet of disordered electrons in a two-dimensional honeycomb lattice that contains two sublattices induced by the interaction of the impurity potentials of two Dirac points. The non-local correlations between the honeycomb lattice and the Dirac points have been quantified by using the Bell function, the uncertainty-induced non-locality, and the concurrence. We have investigated the ability of the lattice-point interaction to create lattice-point non-local correlations under the effects of the band parameter, intravalley scattering processes, and the wave numbers. The generation of lattice-point non-local correlations has been explored when the lattice-point system is initially in the uncorrelated state. We have noticed that the produced Bell-function non-locality and the entanglement exhibit the same oscillatory behavior and satisfy the hierarchy principle. We have analyzed the robustness of the CE, UIN, and MBF non-local correlations by varying the band parameter, the intravalley scattering processes, the wave numbers, and the intrinsic decoherence for an initial maximally correlated lattice-point state. We deduce that the generation and robustness of lattice-point correlations are very sensitive to the honeycomb lattice and Dirac point parameters.
